# Italian guidelines for the use of digital breast tomosynthesis in breast cancer screening programmes: GRADE-ADOLOPMENT of the European guidelines

**DOI:** 10.1007/s11547-026-02186-0

**Published:** 2026-03-05

**Authors:** Francesco Venturelli, Silvia Deandrea, Paola Mantellini, Eugenio Paci, Marco Zappa, Martina Rossi, Massimo Calabrese, Francesca Battisti, Lauro Bucchi, Priscilla Sassoli de’ Bianchi, Francesca Caumo, Carlo Senore, Adriana Bonifacino, Flori Degrassi, Valeria Fava, Elisa Betti, Riccardo Vecchio, Andrea Guida, Gian Paolo Morgano, Livia Giordano, Paolo Giorgi Rossi, Francesco Sardanelli, the Italian mammography screening and diagnosis guidelines Technical/Scientific Committee, Panel and Reviewers, Vittorio Cannatà, Isabella Castellano, Gabriella Dardanoni, Giulio Francolini, Stefania Gori, Carlo Magliocca, Stefania Montemezzi, Stefano Pacifici, Emanuele Torri, Annalisa Trianni, Antonella Pellegrini, Daniela Ambrogetti, Catia Angiolini, Paola Bellardini, Laura Bonvicini, Beniamino Brancato, Stefano Campa, Matteo Capobussi, Eva Carnesciali, Leopoldo Costarelli, Prassede Foxi, Paola Golinelli, Icro Meattini, Marco Moschetta, Paola Mosconi, Loredana Pau, Stella Pedilarco, Francesca Pietribiasi, Marco Rosselli del Turco, Marzia Salgarello, Alfredo Santinelli, Gioele Santucci, Roberto Satolli, Cristian Scatena, Margherita Serra, Mario Taffurelli, Alfonso Frigerio, Luigi Bisanti

**Affiliations:** 1 Epidemiology Unit, Azienda USL-IRCCS Di Reggio Emilia, Reggio Emilia, Italy; 2 Pavia Health Protection Agency, Pavia, Italy; 3 Directorate General for Health, Lombardy Region, Milan, Italy; 4 Institute for Cancer Research, Prevention and Clinical Network (ISPRO), Florence, Italy; 5 Italian League Against Cancer, Tuscany Region Coordinator, Institute for Cancer Research, Prevention and Clinical Network (ISPRO), Florence, Italy; 6 IRCCS, Azienda Ospedaliera Metropolitana (AOM), Policlinico San Martino, Genoa, Italy; 7 Emilia-Romagna Cancer Registry, Romagna Cancer Institute (IRCCS Istituto Romagnolo Per Lo Studio Dei Tumori (IRST) Dino Amadori), Meldola, Italy; 8 Department of Health, Emilia-Romagna Region, Bologna, Italy; 9 IRCCS Istituto Oncologico Veneto IOV, Padua, Italy; 10 Epidemiology and screening unit – CPO - University hospital, Città Della Salute E Della Scienza, Turin, Italy; 11 Fondatrice Fondazione IncontraDonna, Rome, Italy; 12 Associazione Nazionale Donne Operate Al Seno - ANDOS, Rome, Italy; 13 Cittadinanzattiva, Rome, Italy; 14 Department of Public Health, Experimental and Forensic Medicine, University of Pavia; Istituti Clinici Scientifici Maugeri IRCCS, Pavia, Italy; 15 Staff di Direzione Sanitaria, Azienda USL Toscana Centro, Florence, Italy; 16 European Commission, Joint Research Centre (JRC), Ispra, Italy; 17 Lega Italiana Per La Lotta Contro I Tumori (LILT) Milano Monza Brianza, Milan, Italy

**Keywords:** Breast neoplasms, Digital breast tomosynthesis, Europe, GRADE-ADOLOPMENT, Italy, Mass screening

## Abstract

To enhance the quality of organized mammographic screening in Italy, in accordance with national legislation, a multidisciplinary panel of experts applied the Grading of Recommendations, Assessment, Development and Evaluation (GRADE)-ADOLOPMENT approach to adopt or adapt the European Commission Initiative on Breast Cancer (ECIBC) guidelines concerning the use of digital breast tomosynthesis (DBT). As prerequisite conditions for DBT adoption, the panel defines a full extension to women in the 45−74 age range, sufficient technical and professional resources, and an adequate monitoring system. The panel recommends the use of either DBT or digital mammography (DM) for asymptomatic women participating in organized screening programmes. However, it suggests prioritizing DBT in women with high mammographic breast density(classified as BI-RADS class *c* or *d*) when density has been previously assessed with DM. In the case of limited resources, priority in the implementation should be given to women with extremely dense breasts (BI-RADS class *d*). The use of DBT as an additional screening tool alongside DM is not recommended. These guidelines aim to provide a tailored approach for screening women with high mammographic breast density, improving detection while optimizing resource allocation in the context of organized screening. However, these recommendations also apply to the setting of spontaneous screening.

## Background

Mammography screening has been shown to reduce breast cancer (BC) mortality, yet it is not without drawbacks. Undesirable effects are mainly related to false positive results and overdiagnosis, which can lead to overtreatment [1, 2]. In addition, the sensitivity of mammography is suboptimal, limiting its efficacy as a screening tool [3].

Digital breast tomosynthesis (DBT), a *quasi*-three-dimensional imaging technology [4], has been demonstrated to be more sensitive compared to digital mammography (DM) [5–11]. Some studies have reported higher DBT specificity, while others have reported a similar or slightly lower specificity compared to DM [7, 12]. Thus, higher sensitivity could provide the opportunity to improve desirable effects of screening through earlier diagnosis and possibly improved prognosis, but there is a need not to increase one of the main undesirable effects of screening, *i.e.* false positives and overdiagnosis. Of note, the long-term impact of DBT is still unknown, both in terms of overdiagnosis and overtreatment as undesirable effects and better prognosis and reduced mortality as desirable effects.

Recently, DBT has been recommended by the new United States Preventive Services Taskforce (USPSTF) and conditionally suggested by other guidelines [13, 14]. To address existing knowledge gaps, the Italian Ministry of Health funded the MAITA project, a collaborative initiative aimed at investigating the downstream consequences of DBT and assessing all the organizational aspects linked to the introduction of DBT in screening. This project brought together all Italian randomized trials on DBT [15].

The European Commission Initiative on Breast Cancer (ECIBC) released several updates following the first recommendations on DBT, published in 2016 [16]. These updates have progressively supported the use of DBT in screening programmes, starting with women with dense breasts and now extending to all women [14]. The Italian National Centre for Screening Monitoring (Osservatorio Nazionale Screening), in collaboration with the Italian Mammographic Screening Group (Gruppo Italiano Screening Mammografico), launched a process for adopting or adapting of the European recommendations in the Italian screening programmes [17]. This effort follows the GRADE-ADOLOPMENT approach [18], ensuring a rigorous and context-specific approach. The project involves multidisciplinary collaborations, engaging scientific societies that address relevant aspects of BC screening and diagnosis. Its Scientific Committee identified DBT-related key questions as a priority. The panel used the systematic reviews and the Evidence to Decision (EtD) tables prepared by the ECIBC Guidelines Development Group (GDG) for the recommendations released in 2021 [19].

This manuscript presents the Italian recommendations on the use of DBT in BC screening and outlines the process of adopting/adapting the ECIBC recommendations. The recommendations integrate evidence synthesized by the ECIBC with findings specific to the Italian context, particularly insights derived from the MAITA project.

## Methods

### ADOLOPMENT process

In line with Italian law no. 24/2017, the National Centre for Clinical Excellence, Quality and Safety of Care–CNEC of the Italian National Institute of Health–ISS, entrusted Italian scientific societies with producing national guidelines, to be included in the New National Guidelines System (SNLG) database [20]. In 2020, the Italian Osservatorio Nazionale Screening, via the Gruppo Italiano Screening Mammografico, reviewed existing BC screening recommendations and initiated the ADOLOPMENT process to adapt the ECIBC guidelines to the Italian context. Details on this process have been provided in a previous publication [17].

The Italian GDG prioritized four ECIBC recommendations on the use of DBT for evaluation within the Italian *ADOLOPMENT* process. This process involves the adoption, adaptation, or de novo development of existing recommendations to align with national requirements and contexts [18].

### Adoption or adaptation of ECIBC recommendations

Below are reported the four healthcare questions prioritized by the ECIBC on the use of DBT [14].

- Use of DBT in women at average risk of developing BC:
  - Should screening using DBT vs. DM be used in organized screening programmes for early detection of BC in asymptomatic women?
  - Should screening using DBT in addition to DM vs. DM alone be used in organized screening programmes for early detection of BC in asymptomatic women?
- Use of DBT in women with high mammographic breast density:
  3. Should DBT vs. DM be used in organized screening programmes for early detection of BC in asymptomatic women with high mammographic breast density detected in a previous screening exam?
  4. Should tailored screening with additional DBT vs. no additional DBT be used in organized screening programmes for early detection of BC in asymptomatic women with high mammographic breast density detected for the first time with DM in screening?

The Italian GDG decided to adapt Questions 1 and 3 while adopting Questions 2 and 4. The rationale for the adoption of recommendations related to questions 2 and 4 was that they were not considered relevant to the current Italian context. Therefore, an adaptation process would not be useful. For the adopted recommendations, the original EtDs were obtained, and the recommendations were translated into Italian. For the adapted recommendations, the Italian GDG acquired the EtD from the ECIBC GDG and revised the information within the EtD domains by incorporating contextual data and an updated literature.

For the two adapted questions, the systematic assessment of the information to be included was led by the PICO^1^ Responsible Unit, a subgroup of panellists involved in providing input to the Evidence Review Team on evidence and contextual factors, summarizing findings, and presenting the updated EtD to the panel during the plenary sessions. The GRADEpro Guideline Development Tool was employed as a digital support during the entire process (https://gradepro.org).

Within the evidence updating process for adapting Question 1, the PICO Responsible Unit proposed that the panel split the question by age (*i.e.* distinguishing women aged 45–49 from women aged 50–74).

### PICO framework of adapted clinical questions

Questions 1 and 3 compared the same intervention (DBT) with the same comparator (DM) in two different populations. In Question 1, the population is represented by all asymptomatic women in the target age for BC screening. In contrast, Question 3 focuses specifically on women with high mammographic breast density identified during a previous screening examination.

The outcomes prioritized by the ECIBC GDG were the same for both Questions 1 and 3: BC mortality, BC stage, BC detection, interval BC rate, recall for assessment, quality of life, other-cause mortality, and adverse effects, including radiation exposure, radiation-induced cancers related to radiation dose, overdiagnosis-related adverse effects, and false positive-related adverse effects.

### Evidence sources by EtD domain

For the adapted healthcare questions, the Evidence Review Team produced an updated EtD starting from the EtD provided by the ECIBC GDG within the GRADEpro platform of the European Commission. The methods used to integrate the EtD domains with contextualized and updated evidence are reported in Table 1.

**Table 1:** Additional evidence provided for the ADOLOPMENT Evidence-To-Decision (EtD) domains and evidence sources used in addition to the ECIBC EtD domains

EtD domain	Evidence sources
Desirable effects	• Update of the literature review up to July 2022 • Context specific data collected within the pilot study conducted in the Autonomous province of Trento, Italy [21, 22] • Results of the trials included in the MAITA project [10, 11, 15, 23]
Undesirable effects	
Resources required	• Budget Impact Analysis informed by the trials results conducted within the MAITA project [24]
Cost effectiveness	• Cost-consequence analysis conducted within the MAITA project [24] • Re-evaluation of the models included in the evidence provided by the ECIBC for relevance and consistency with context-specific and trial-informed parameters
Equity	• Structured interviews to key persons within the MAITA project [25]
Acceptability	• Structured interviews to key persons within the MAITA project [25] • Survey on awareness and acceptability towards DBT by 45-year-old women participating to mammographic screening at ISPRO, Florence • Context specific data collected within the pilot study conducted in the Autonomous province of Trento, Italy. [21, 22]
Feasibility	• Structured interviews, surveys, and systematic reviews on organizational impact, technical requirements, reading and acquisition times of DBT in screening conducted within the MAITA project [25]

The studies included in the ECIBC literature review and the newly included studies were also assessed to extract data stratified by age or breast density, when available.

The following strategy was used for synthesizing the literature review update and the MAITA trials. If the findings of the included studies aligned with those reported in the ECIBC review, they were documented as additional evidence without being incorporated into the meta-analysis. However, if discrepancies were identified, a new meta-analysis was conducted, incorporating both the new studies and the trials included in MAITA.

### Certainty of evidence

The panel, according to the GRADE methodology, reassessed the certainty of evidence for desirable and undesirable effects, and for required resources.

### External peer review

The revised recommendations and supporting documents underwent external peer review, were assessed for compliance with the AGREE II checklist criteria [26], and were finally approved by the reviewers of SNLG before publication on the Italian guidelines database [27].

## Results

### Results in women at average risk of developing BC (Question 1)

#### Desirable and undesirable effects

The update of the ECIBC literature review up to July 2022 identified six studies to be included, of which five studies provided evidence for Question 1 [21, 28–31] and two studies for Question 3 [31, 32]. The results of the trials conducted as part of the MAITA project were also incorporated, some of them even prior to publication in peer-reviewed journals [10, 11, 15].

The new results were reported as additional evidence for both Questions 1 and 3. The new evidence on detection rates was consistent with the findings included in the ECIBC literature review. Results on the recall rate showed heterogeneity among studies and among centres within studies [15]. This supports previous research on the specificity of radiologists’ recall, which has been shown to be more influenced by the local practices than by their sensitivity [33, 34]. The results of the MAITA trials provided evidence on cumulative incidence on two screening rounds not included in the EtD used by the ECIBC. The updated evidence included for the desirable and undesirable effects criteria in the EtD for Question 1 is summarized in Table 2.

**Table 2:** Data from studies already included in the ECIBC literature review

Outcomes	Study	Number of participants (studies)	Effect measure	Relative effect (95% CI)	Risk difference with screening using DBT
Question 1—Women at average risk of developing BC					
Breast cancer detection	TOSYMA Screening Trial [30]	99,689	Odds ratio	1.48, 95% CI 1.25 to 1.75	2.3 per 1000; 95% CI 1.3 to 3.3
	Houssami, 2021 [29]		Pooled incidence rates per 1000	9.03; 95% CI 8.53 to 9.56	/
	MAITA	114,000	Relative risk	1.51 (95% CI 1.30 to 1.75)	0.79% DBT + DM vs. 0.52% DM
BC stage (advance cancer detection rate)	Kerlikowske 2022 [31]	504,150	Screening outcomes per 1000 examinations	0.36 DBT vs. 0.45 DM	− 0.09; 95% CI − 0.18 to − 0.01
Interval cancer	Kerlikowske 2022 [31]	504,150	Screening outcomes per 1000 examinations	0.57 DBT vs. 0.61 DM	− 0.04; 95% CI − 0.14 to 0.06
	Houssami 2021 [29]	129,969 DBT and 227,882 mammography	Pooled rates per 1000 screens	1.56/1000 DBT vs. 1.75/1000 DM	− 0.15/1000; 95% CI − 0.59 to 0.29
	To-Be [28]	14,848	Rates per 1000 screened woman	1.4/1000 DBT vs. 2.0/1000 DM	
			Relative risk	0.69 (95% CI 0.39 to 1.22)	
False positive recall for assessment	MAITA	114,000	Relative risk	1.15 IC 95% 1.09 to 1.22	5,05% vs. 4,43%
	Kerlikowske 2022 [31]	504,427	Rates per 1000 screens	66.2 DBT vs. 83.4 DM	− 17.2; 95% CI − 25.2 to − 9.2
Overdiagnosis	MAITA	104,000	Cumulative incidence	Potential overdiagnosis: 2.5/1000 (95% CI 1.1–3.9) for screened women	
	Houssami 2021 [29]	129,969 DBT and 227,882 mammography	Pooled rates per 1000 screens	Potential overdiagnosis	
Radiation dose	STORM-2 [35]	1,208	MGD, mean glandular dose (mGy)	CC DM = 1.366 CC DBT = 1.858 MLO DM = 1.374 MLO DBT = 1.877	/

#### Results stratified by age

A total of 7 studies, including those from the literature review and from the MAITA trials, reported results stratified by age (40 or 45–49 or 50–69 years).

The detection rate results, stratified by age, were inconsistent across the five studies reporting data on the detection rate. Two studies showed a similar increase in detection rate with DBT compared to DM across both age strata [36, 37]. In contrast, two other studies reported a higher increase in detection rate with DBT versus DM in women aged 40–49, compared to those aged 50–69 [38, 39]. Conversely, the MAITA trials showed a higher increase in detection rate with DBT vs. DM in women aged 50–69 compared to those aged 45–49 [15].

Regarding the recall rate, five of the seven studies reporting results on age-stratified data showed a lower recall rate with DBT than with DM [36, 38–41], while two studies reported a higher overall recall rate with DBT [15, 37]. No differences in relative effects between age strata were reported, apart from two studies. The first reported a slight increase in recall rate only in women aged 50–59 and no difference in younger women [37], while the second one showed a slightly larger reduction in recall rate in women aged 40–49 than in those aged 50–59 and 60–69 [41].

Data on interval cancer stratified by age were available from one study [38] and from the MAITA trials (unpublished data). No differences in interval cancers after a negative DBT or DM in any age group were observed.

Data on cumulative incidence after two screening rounds, stratified by age, were only available from the MAITA trials. The increase in cumulative incidence in the DBT+DM arm compared to the DM arm was similar between women aged 40–49 and 50–69, relative risk 1.10 (95% CI 0.80 − 1.51) and 1.13 (95% CI 1.00 − 1.27), respectively. Despite this, the results were inconsistent between the two larger trials. RETomo reported an increase in cumulative incidence only in women aged 50–69 with relative risk of 0.93 (95% CI 0.73–1.20) and 1.12 (95% CI 1.00–1.24), respectively, while Proteus reported an increase in all age strata, with a larger effect in women aged 40–49 compared to those aged 50–69, with a relative risk of 1.17 (95% CI 0.94–1.47) and 1.08 (95% CI 1.00–1.17), respectively (unpublished data).

Overall, evidence stratified by age did not suggest different desirable and undesirable effects of DBT vs. DM for both women aged 40–49 and women aged 50–69. Consequently, the Panel decided not to issue a separate recommendation for women aged 45–49.

#### Results in women with high breast density (Question 3)

Two studies included in the literature review reported results for women with high breast density. The MAITA trials provided data on breast density for 24,495 women recruited by the RETomo study, of whom 11,948 were categorized as having high breast density (BI-RADS class* c* or *d*) [10, 15, 31, 32].

*Detection rate.* The RETomo results showed a higher detection rate for the DBT+DM group compared to DM alone in all the breast density strata. The relative risk was 1.6 (95% CI 1.0–2.5), similar to the 1.7 (95% CI 1.2–2.3) observed in all women [10]. The study by Kerlikowske et al. [31] reported similar results across breast density strata, with the only exception of a higher detection rate of stage I cancers in the DBT group compared to DM in women with breast density BI-RADS category *b* (scattered fibroglandular). The stage I screen-detected invasive cancer detection per 1000 exams was 3.74 (95% CI 3.54–3.94) in the DBT and 3.30 (95% CI 2.98–3.65) in the DM group, with a difference of 0.44 (95% CI 0.07–0.81) [31]. The TOSYMA trial showed a higher detection rate of invasive cancer in the DBT group compared to the DM group in women with high breast density. In particular, for women with breast density category BI-RADS *d*, the risk difference was 5.8 per 1000 screened women (odds ratio 3.8, 95% CI 1.5–11.1), while for women with breast density category BI-RADS *c* or *d* the risk difference was 2.7 per 1000 screened women (OR 1.48, 95% CI 1.17–1.87) [32].

*Interval cancers and advanced BCs.* In the RETomo trial, in women with breast density BI-RADS *c* or *d* the interval cancer rate was similar between the two arms: 0.23% (*n* = 14 cancers) in the DBT+DM and 0.25% (*n* = 15 cancers) in the DM group (relative risk 0.9, 95% CI 0.5–1.9) [10]. The incidence of advanced BCs will be assessed within the MAITA trials when the 4.5-year follow-up of two screening rounds will be completed. No differences in the interval cancer rate, as well as in the incidence of advanced BCs between the two arms in any breast density stratum, were found in the study by Kerlikowske et al. (2022) [31].

One panellist reported results from a one-arm prospective pilot study performed in Verona, Italy, in which women underwent one round of screening with DBT+synthetic 2D views [42]. At the following round, the proportion of cancers stage II or higher was 14.5% (19 of 131 cancers) in women screened with DBT+synthetic 2D views and 8.5% (five of 59 cancers) in women screened with DM, both of which were lower than the proportion in the historical control group screened with DM (30 of 110 cancers, 27.3%) (*p* ≤ 0.01).

*False positive recall rate.* The RETomo trial showed a similar recall rate in the DBT+DM group (4.5%, *n* = 266 recalls) compared to the DM group (4.8%, *n* = 286 recalls) in women with breast density BI-RADS categories *c* or *d* (relative risk 0.9, 95% CI 0.8–1.1). Similar results were reported for the false positive recall rate in the DBT+DM group (3.6%, *n* = 217 false positive recalls) and the DM group (4.3%, *n* = 255 false positive recalls), resulting in a relative risk of 0.9 (95% CI 0.7–1.0). Thus, the relative effects were slightly lower for women with breast density BI-RADS *c* or *d* than the one observed overall (relative risk 1.15 95% CI 1.09–1.22) [10]. Kerlikowske et al. reported similar false positive recall rates between the two arms, with the only exception for women with breast density categories BI-RADS *a* (almost entirely fatty) and *b* that showed lower rates in the DBT arm compared to DM [31]. The TOSYMA trial reported increasing false positive recall rates at increasing breast density categories, even though there was no difference between the DBT and DM arms in any density stratum [32].

*Overdiagnosis.* The results of the second screening round for women with breast density categories BI-RADS *c* or *d* recruited within the RETomo trial showed a lower detection rate in the DBT+DM arm (0.33%) compared to the DM arm (0.60%) with an incidence rate ratio of 0.56 (95% CI 0.32–0.98). The two-round cumulative incidence was similar between the two arms, with 88 cancers detected in the DBT+DM group and 83 cancers in the DM group, and an incidence rate ratio of 1.06 (95% CI 0.79–1.43) [10].

The panel expressed concerns about considering the increased detection rate with DBT as a desirable effect for the general population, due to the absence of a reduction in interval cancers and the observed excess in cumulative incidence. In contrast, for women with dense breasts, the panel evaluated positively the increase in detection rate obtained by DBT, interpreting these data as a more probable potential benefit (*i.e.* early diagnosis of clinically relevant lesions) than as overdiagnosis leading to overtreatment. This positive assessment was supported by the reduced incidence of cancers in the second screening round and the absence of excess cumulative incidence across both rounds. The panel also considered a modest level of indirectness in the use of mixed protocols (DBT+DM and DBT+synthetic 2D), as the number of lesions detectable by DM but not by DBT was negligible in most studies [5, 23]. Consequently, the downstream consequences of screening could not be influenced by the treatment of these lesions.

#### Results on questions exploring the impact of adding DBT to DM (Questions 2 and 4)

Regarding Questions 2 and 4 (*i.e.* adding DBT to DM in the same screening round), the Italian GDG adopted the recommendations issued by the ECIBC GDG in 2021. In this version of the recommendations, using both technologies (i.e. DBT+DM) was never suggested. In 2024, the ECIBC opened for adding DBT to DM only in women with high mammographic breast density detected for the first time with DM in screening [14]. However, due to concerns related to the double radiation exposure, this option is not suggested in the Italian ADOLOPMENT.

#### Resources and cost effectiveness

Information on resources required included in the ECIBC EtD was integrated with the results of the budget impact analysis conducted within the MAITA project, informed by parameters estimated from the MAITA trials [24]. This analysis estimated the cost of substituting DM with DBT in a two-round horizon (*i.e.* 4 years) of biennial BC screening in Italy, considering four different scenarios (*i.e.* DBT for all women, DBT for women aged 45–49 only, DBT for women with breast density BI-RADS *c* or *d,* or only *d*).

The budget impact analysis estimated an overall increase in screening costs of 20% with the introduction of DBT for all women eligible for BC screening. In the targeted scenarios, the cost increase was 3.2% with the introduction of DBT for women aged 45–49 only and 1.4% for women with density BI-RADS *d* and 10.7% for women with density *c* or *d*.

These results were consistent with the estimates provided by two studies from the Netherlands, which were included upon the suggestion of the PICO Responsible Unit. The first level screening cost per screened woman estimated by the first study ranged from 80 to 96 € with DBT and was 64 € with DM [43]. The second study estimated was 96€ with DBT and 66€ with DM [44]. The To-Be study estimated an increase in costs of 8.5 € per screened woman with DBT compared to DM, a lower level than the estimates from previous studies [45]. Estimates from the pilot implementation of DBT in the Autonomous Province of Trento reported a cost per screened woman ranging from 59€ to 84€, depending on the organizational model [22].

Considering this evidence, the Italian panel observed that the increase in costs would be smaller in the centres in which the recall rate was reduced, like in the Trento pilot, than in centres where the recall would be unaffected or increased. Noteworthy, a reduction in recall rate was observed in Trento, but was not observed in any of the studies included in the MAITA project. Nevertheless, the Italian panel highlighted that the main driver of costs is the increase in reading time, which is independent of the recall rate and could represent a relevant obstacle when considering the shortage of reading radiologists (see below).

Given the consistency between the results of the estimates of cost increase between the MAITA project and previous studies from Norway and the Netherlands, the Italian panel decided to confirm the judgement on resources provided by the ECIBC panel of moderate costs. The cost increase in the density-based scenario would be lower and strictly dependent on the decision of the BI-RADS categories to be invited to DBT (10.7% increase for BI-RADS *c* or *d* categories, and 3.2% increase for the BI-RADS *d* category).

Within the MAITA project, it was decided to perform a cost-consequence rather than a cost-effectiveness analysis. This choice was motivated by the uncertainty of downstream consequences, particularly by the absence of interval cancer reduction, while all previous cost-effectiveness models assumed a reduction in interval cancers as a consequence of increased sensitivity [24, 43–45],

The Italian panel confirmed the judgement of the ECIBC panel on cost-effectiveness in favour of the DM for all women, given the same judgement on costs and a balance of effects not in favour of DBT. On the contrary, given a balance of effects in favour of DBT and lower costs in the density-based scenario, the cost-effectiveness was judged in favour of DBT for women with high breast density.

#### Equity, acceptability, and feasibility

No recent studies reporting data on the impact on equity have been identified in the literature. Within the MAITA project, structured interviews with key persons in screening programmes highlighted that 60% of respondents did not consider a plausible impact on equity likely. Nevertheless, in their opinion, DBT could increase women’s adherence to organized screening, as this examination is already offered in private or clinical breast imaging settings [25]. DBT, therefore, could make organized screening more attractive, especially for women with higher socio-economic status, who may favour private practice instead of public organized screening. However, it is necessary to consider a potential proportion of users who may refuse the examination due to concerns about increased radiation exposure. Furthermore, according to the panel, the lower sustainability of a program adopting DBT could lead to a reduction in screening invitation coverage, mainly due to increased reading times against the shortage of screening reading radiologists in the public healthcare system.

A study on the acceptability of DBT by radiologists [46] has highlighted several relevant themes related to the context of organized screening, such as the use of synthetic 2D images or the difficulty in accessing stereotactic biopsy procedures with DBT. Moreover, from the experience of the RETomo trial, lower acceptability of DBT+DM was reported due to increased compression times, a concern that has been partially overcome using synthetic 2D images, despite DBT being a longer examination. Nevertheless, adherence to trials for the MAITA project was reported to be above 65%.

Finally, concerning feasibility, whilst a systematic literature review was not conducted, several themes were included in the context of the MAITA project [25]. Italy has one of the highest numbers of mammography units per capita in Europe. A report from Confindustria based on data collected by the Italian Ministry of Health shows that, at the end of 2021, of 2039 mammographic units in Italy, 33.4% were still conventional (not digital), while the mean age of the digital units was 4.8 years [47]. Equipment renewal during the last three years could have increased the percentage of digital units and potentially reduced their mean age. At any rate, the introduction of DBT should work as a final push towards a complete decommissioning of conventional units. However, the adoption of DBT requires, initially, doubled quality control times, greater memory for image storage and transmission, as well as increased organizational complexity. Regarding this last point, several clinical trials within organized programmes have demonstrated that the introduction of DBT is feasible, at least in contexts with robust and flexible management information technology systems. Lastly, radiology technicians have highlighted the need for specific training for all personnel, particularly those involved in front-office activities, to correctly answer women’s doubts and questions.

#### Final recommendations and summary of judgements for EtD Question 1 and Question 3

The Italian recommendations for the four prioritized questions are shown in Table 3. The summary of judgements on adapted recommendations from the ECIBC and Italian GDG is reported in Table 4.

**Table 3:** Synopsis of the recommendations and decisions of the Italian Guidelines Development Group on the adoption or adaptation of ECIBC guidelines according to the GRADE-ADOLOPMENT approach

	Screening with DBT vs. DM	Screening with DBT plus DM vs. DM
Asymptomatic women	Q1: For asymptomatic women with an average risk of BC the Italian panel suggests using either DBT or DM in the context of an organized screening programme (conditional recommendation, low certainty of the evidence)	Q2: For asymptomatic women with an average risk of BC, the Italian Panel adopts the recommendation of the ECIBC's Guidelines Development Group (GDG) and suggests not using both DBT and DM in the context of an organized population-based screening programme (conditional against, very low certainty of the evidence)
Women with high breast density	Q3: For asymptomatic women with high mammographic breast density detected in previous screening exams, the Italian panel suggests using DBT over DM in the context of an organized population-based screening programme (conditional recommendation, low certainty of the evidence)	Q4: For asymptomatic women with high mammographic breast density detected for the first time with DM, the Italian Panel adopts the recommendation of the ECIBC's GDG and suggests not implementing tailored screening with additional DBT in the context of an organized population-based screening programme (conditional against, very low certainty of evidence)

**Table 4:** Changes in judgements for adapted recommendations by the Italian panel to the source guidelines (in light grey, the criteria that were adapted to the Italian context by the panel)

Etd criteria	Judgement on Q1		Judgement on Q3	
	original	ADOLOPMENT	original	ADOLOPMENT
Problem	Yes	Yes	Yes	Yes
Desirable effects	Moderate	Moderate	Moderate	Moderate
Undesirable effects	Small	Moderate	Small	Small
Certainty of evidence	Very low	Low	Very low	Low
Values	Important uncertainty or variability	Possibly important uncertainty or variability	Possibly important uncertainty or variability	Possibly important uncertainty or variability
Balance of effects	Probably favours the intervention	Does not favour either the intervention or the comparison	Probably favours the intervention	Probably favours the intervention
Resources required	Moderate costs	Moderate costs	Moderate costs	Moderate costs
Certainty of evidence of required resources	Low	Moderate	Low	Moderate
Cost-effectiveness	Probably favours the comparison	Probably favours the comparison	Probably favours the intervention	Probably favours the intervention
Equity	Varies	Varies	Varies	Varies
Acceptability	Varies	Varies	Varies	Probably yes
Feasibility	Varies	Probably no	Varies	Varies

Recommendations are presented in the format: strength and direction of recommendation; in parentheses, the certainty of evidence. The number corresponds to the original questions. DBT: Digital breast tomosynthesis; DM: Digital mammography.

## Discussion

### Summary statement

Using the GRADE-ADOLOPMENT approach, four recommendations on the use of DBT from the ECIBC Guidelines on Breast Cancer Screening and Diagnosis were adopted or adapted to the Italian national context. In particular, two recommendations were adapted; for each of them, an EtD table was provided using evidence from the source guidelines, evidence solicited by the panel, and context information from Italy. For BC screening, the Italian panel specifically suggests using either DBT or DM in organized screening programmes. The use of DBT is suggested over DM for women who have high mammographic density (BI-RADS density class *c* or *d*) found in prior screening exams, with priority given to women with extremely dense breasts (BI-RADS density class *d*). Double radiation exposure with DBT+DM is never suggested as an option.

### Comparison with other guidelines

In 2024, the ECIBC updated the recommendations on DBT, changing the statement for women at average risk of developing BC from a recommendation for either DM or DBT to a conditional recommendation in favour of DBT. The update was based on a substantially different body of evidence compared to the previous one. In the ECIBC update, the publication of the follow-up results of the To-Be trial, which, to date in 2024, is the only trial testing DBT+synthetic 2D views providing data on interval cancers and reporting results of the following screening round, led to the decision to exclude the indirect evidence on interval cancers and following round coming from trials using DBT+DM, i.e. from the two Italian trials. The Italian ADOLOPMENT included the To-Be follow-up results in the summary of findings and the EtD. Nevertheless, the panel decided to consider also the indirect evidence of a small, if any, impact on interval cancers. This choice is reasonable because despite To-Be found a nonsignificant reduction of interval cancers after DBT+synthetic 2D, there is no rationale for a higher sensitivity of DBT+synthetic 2D than DBT+DM. Furthermore, the To-Be trial performed DBT at the following round in both arms, therefore it cannot give any insight into the impact of DBT on overdiagnosis, since all women at the end of the study received at least one DBT.

The USPSTF, in April 2024, published new recommendations in favour of DBT [13]. The decision was based mostly on the reduction of the harms of screening due to false positives. The impact on recall and false positives showed to be scarcely generalizable across studies and countries. In the Italian trials, we did not observe an overall decrease in recall, but results were heterogeneous across centres. Therefore, it is not surprising that the judgement about the false positive is different in guidelines developed in different contexts.

### Implementation considerations and implications for practice

The panel highlights that the present recommendations, despite being developed with the scope of informing decision-making in the organized programmes, should also be considered in the setting of spontaneous screening. As prerequisite conditions for DBT adoption, the panel defines a full extension to women in the 45–74 age range [17], a sufficient number of DM units equipped with DBT, sufficient resources to address increased reading times, and an adequate monitoring system. The panel emphasizes that during the phase of extending the screening target age from 50–69 to 45–74, resources should not be diverted to the introduction of DBT on a large scale. Regarding the specific subgroup of women aged 45–49 years, no differential recommendation is expressed concerning DBT or DM use. Conversely, for the subgroup of women with dense breasts of any of the age strata, the panel acknowledged that radiologist resources needed could be reasonable and available in some contexts, thus overcoming the critical issue related to increased reading times. These recommendations allow an implementation of DBT based on breast density, a characteristic influencing women’s risk and test accuracy. If a screening program adopts this strategy, effective communication is important to explain why one woman may benefit more than another from DBT. Correct information should include understandable information on how test accuracy changes by breast density, and the rationale for proposing different tests. A large survey in the US, included among the evidence assessed by the ECIBC GDG, reported that 60% of women participating in screening overestimated the risk of radiation exposure. [48] Accordingly, ECIBC GDG, when affording DBT-related clinical questions, recognized the importance of addressing inappropriate concerns about radiation dose in programmes using the DBT plus DM combination. The ECIBC GDG also recognizes the importance of informing women and healthcare professionals about the risks of radiation in the context of the potential benefits of screening. [2]

The panel also highlights that artificial intelligence (AI) technology could play a pivotal role in addressing resource challenges. AI-driven triage systems and workflow optimization could help streamline processes and improve resource allocation, potentially enhancing the feasibility of integrating DBT into routine screening practices. Population-based prospective studies have proven that AI can safely reduce the number of human readings required for screening by 35% to 50%. [49–51] Unfortunately, all of these studies have been conducted using 2D mammography. Recent retrospective studies of large archives of images [52], as well as demonstration studies [53], have shown that AI systems can achieve comparable results when working with DBT images [54]. Forthcoming activities of the Italian group include the ADOLOPMENT of the ECIBC recommendations on AI in the reading of screening mammograms. The collection of mammographic density information requires shared and reproducible assessment methods and would be utilized both for screening program management and its monitoring and evaluation. The recommendation refers to breast density as classified in the original studies, mostly classified by human readers according to BI-RADS density class *c* or *d*. The ECIBC GDG, based on an ad hoc review of the literature, analysed the issue of how breast density should be classified and the limits of the existing systems. The GDG identified as the first research priority “examining the classification of mammographic breast density and standardization of the classification systems used for breast density, including technology for the automation of determining breast density. Research should also aim at establishing the appropriate density threshold for additional imaging”. The need for a reproducible classification of breast density has also been highlighted by the Italian working group in the implementation considerations. Computer-based tools for the automated quantification of breast density may increase the reproducibility of density evaluation.

### Implication for research

The panel recommends carefully evaluating the emerging results from ongoing trials in Italy and across Europe. Furthermore, the implementation of DBT in organized screening programmes will necessitate data collection on processes and outcomes, enabling the production of new evidence from current practice; this process will be facilitated by stratification variables such as mammographic density. Implementation conditions that may enhance sustainability and feasibility across all contexts should be investigated; among these, it is particularly promising the use of AI-based algorithms to triage images requiring two human readings or only one human reading [49, 50], or even no human reading, when negative predictive values near 100% will be reached for well-defined subgroups of patients/mammograms. Systematic collection of mammographic breast density in women regularly attending screening before recommendations adoption would enable the evaluation of optimal implementation strategies, also considering the relationship with age, and prioritization decisions. Finally, it will be valuable to gather information on potential communication and organizational challenges, particularly when screening programmes decide to adopt a tailored strategy for women with dense breasts.

## CONCLUSIONS

The Italian recommendations suggest that DBT (meaning DBT plus synthetic 2D views) can be used in the screening practice, particularly in women with dense breasts. They acknowledge feasibility issues due to a large increase in required resources upfront to uncertain health benefits. As prerequisite conditions for DBT adoption, the panel defines a full extension to women in the 45–74 age range [17], sufficient technical and professional resources, and an adequate monitoring system. The panel recommends the use of either DBT or digital mammography (DM) for asymptomatic women participating in organized screening programmes. The use of DBT is suggested over DM for women who have high mammographic density (BI-RADS density class *c* or *d*) found in prior screening exams, with priority given to women with extremely dense breasts (BI-RADS density class *d*). The direction and the strength of the recommendations are identical to those issued by the ECIBC in 2021, despite the evidence being substantially updated. Regarding adding DBT to DM in the same screening round, the Italian ADOLOPMENT adopted the previous recommendations from ECIBC (2021) in which double exposure was never suggested. In 2024, the ECIBC opened for adding DBT to DM only in women with high mammographic breast density detected for the first time with DM in screening [14]. This option is not suggested in the Italian ADOLOPMENT.

**Fig.1:**
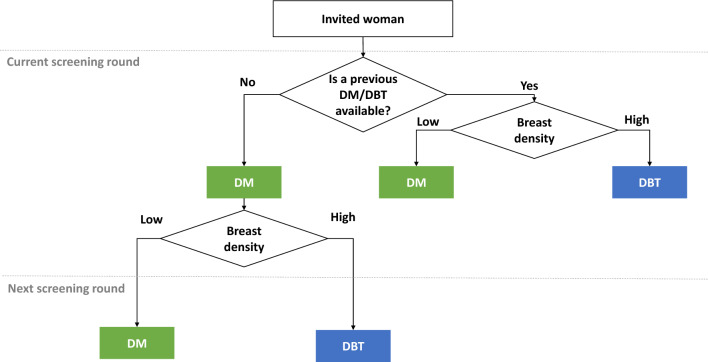
Flow chart showing the eventual application of tailored screening, stratified by breast density. The recommendations for the use of DBT have different directions according to breast density. This situation may open to a tailored implementation of DBT in screening programmes. For women who approach the programme for their first screening round and without any recent breast DM or DBT, the programme will perform a DM. In the following rounds, the programme will offer a DBT or DM on the basis of the breast density assessed at the first round: in case of high mammographic density (BI-RADS D or C+D) the woman will be offered a DBT; in case of low density (A+B+C or A + B), the woman will be offered a DM. For women who are at the following screening rounds, the screening programme can assess the breast density in stored available images and offer DBT or DM according to breast density. This approach requires screening archives linked to RIS-PACS, a condition rarely present in an opportunistic setting

These recommendations open to a tailored approach for screening women with high mammographic breast density, improving detection while optimizing resource allocation in the context of organized screening. However, they also apply to the setting of spontaneous screening. To support the eventual application of tailored screening, stratified by breast density, a flow chart is provided in Fig. 1.

## Footnotes

1 The PICO acronym is used to answer health care-related questions in an evidence-based framework. It stands for: P = Patient, Problem, or Population; I = Intervention; C = Comparison, Control, or Comparator; O: Outcome(s).
